# Ameliorative impacts of *Glycyrrhiza glabra* root extract against nephrotoxicity induced by gentamicin in mice

**DOI:** 10.1002/fsn3.2183

**Published:** 2021-05-22

**Authors:** Mohamed A. Nassan, Mohamed M. Soliman, Adil Aldhahrani, Fayez Althobaiti, Adel Q. Alkhedaide

**Affiliations:** ^1^ Department of Clinical Laboratory Sciences Turabah University College Taif University Taif Saudi Arabia; ^2^ Department of Pathology Faculty of Veterinary Medicine Zagazig University Zagazig Egypt; ^3^ Department of Biochemistry Faculty of Veterinary Medicine Benha University Benha Egypt; ^4^ Biotechnology Department College of Science Taif University Taif Saudi Arabia

**Keywords:** Bax, Cox2, gentamicin, *Glycyrrhiza glabra*, Nrf2

## Abstract

Gentamicin is an effective antibiotic that has been used worldwide for many years. While considered an essential medicine by the WHO, gentamicin can also lead to severe kidney damage. This study explored the ameliorative effects of *Glycyrrhiza glabra* root extract on gentamicin‐induced renal injury in mice. Four groups of *n* = 7 mice were used: (a) control; (b) *G. glabra*‐only; (c) gentamicin‐only; and (d) gentamicin plus *G. glabra*. Kidney samples were tested for: antioxidant enzyme activity (superoxide dismutase [SOD] and glutathione peroxidase [Gpx]); expression of HO‐1 and nuclear factor erythroid 2‐related factor 2 genes; expression of Cox‐2 and Bax; cytokine levels (IL‐1β, and IL‐6); histopathological anomalies; and standard renal functional component levels (creatinine, urea, and blood urea nitrogen). The effects of gentamicin were generally reversed or normalized following treatment with *G. glabra* root extract. Gentamicin decreased Gpx and SOD parameters and increased IL‐1 β and IL‐6 levels, but these returned to normal in the *G. glabra*‐treated group. Gentamicin upregulated tissue levels of Cox‐2 and Bax, and downregulated HO‐1 and Nrf‐2 expression but again, and these levels returned to normal in the group treated with *G. glabra*. Mice that had received gentamicin exhibited acute renal blood vessel congestion, focal interstitial round cell aggregation, and hydropic degeneration of renal tubular epithelium. However, those that had also received *G. glabra* showed a normal histopathology. Findings from this study indicate that in mouse models, gentamicin‐induced kidney damage can be reversed or ameliorated by administering *G. glabra*, so it can be considered as an effective complimentary therapy.

## Graphical abstract

This manuscript discusses the effect of *Glycyrrhiza glabra* root extract against nephrotoxicity induced by gentamicin in mice with evaluation of real‐time pcr gene expressions of nuclear factor erythroid 2‐related factor 2 and HO‐1 and immunohistochemical examination of COX‐2 and Bax together with evaluation of kidney function parameters and cytokines (IL‐1 and IL‐6 levels) with examination of antioxidant parameters (Gpx and SOD).

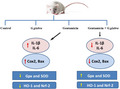

## 1 INTRODUCTION

The kidneys help to control the volumes of various bodily fluids by extracting waste products from the bloodstream to be excreted as urine. In his role, they are particularly vulnerable to xenobiotic nephrotoxic agents, such as drugs or environmental toxins, which can cause a range of damaging effects (El‐Tantawy et al. 2013). Two major players involved in kidney damage and failure are oxidative stress and inflammation responses (Boozari & Hosseinzadeh, 2017). Consequently, a great deal of research is being undertaken to explore the hepatoprotective effects of various compounds. Experimental models for this research include the aminoglycocide nephrotoxicity model (Casanova et al., 2017). One common and widely used aminoglycocide antibiotic is gentamicin (Kopple et al., 2002), which is effective in treating potentially deadly gram‐negative infections. The antibiotic kills bacterial cells by binding to bacterial ribosomes and thereby interrupting protein synthesis. However, side effects of gentamicin include nephrotoxicity and ototoxicity, so its clinical use must be carefully controlled (Sun et al. 2018). Of interest to our study are the nephrotoxic mechanisms of gentamicin, which include oxidative stress and apoptosis.

*Glycyrrhiza glabra* (common name: licorice) is a herb commonly found in Western Asia and Southern Europe. It has antibacterial, antiviral, anti‐inflammatory, and antioxidative properties, as well as detoxifying and hepatoprotective effects (Yu et al., 2015). The plant is effective in treating several common illnesses, including cold and cough symptoms, and kidney and intestinal disorders. Anti‐cancer properties have also been reported (Parlar et al., 2020). It has been used to treat a wide range of other health conditions, including liver disorders, sore throats, gastric ulcers, dyspepsia, asthma, bronchitis, rheumatoid arthritis, and Addison's disease, and has been used as a laxative, an expectorant, and an antitussive (Huo et al., 2011).

The plant extract contains multiple antioxidant and anti‐inflammatory phytochemicals and is reported to protect against hepatotoxic treatments for rheumatoid arthritis (Tan et al., 2018), although this effect is not fully understood. Key biologically active compounds in *G. glabra* include saponins and flavonoids such as liquiritin, liquiritigenin, liquiritin apioside, isoliquiritigenin, glabridin, and glycyrrhizin (Kamei et al., 2003). Free radical‐associated oxidative stress in kidney tissues can be reduced by *G. glabra* (Yokozawa et al., 2005), as can hormone or toxicity‐related carcinogenesis (Mori et al., 2001).

Given the view that natural remedies for nephrotoxicity are considered to be safe and effective, the present study set out to explore the effects of *G. glabra* extract in protecting against nephrotoxicity caused by gentamicin intoxication in male mice.

## 2 MATERIALS AND METHODS

### 2.1 Kits and chemicals

Superoxide dismutase (SOD), glutathione peroxidase (GPX), blood urea nitrogen (BUN), and urea kits were sourced from Biodiagnostic; agarose and ethidium bromide from Sigma‐Aldrich; RNA reverse transcription enzyme and the DNA ladder (100 bp) from MBI, Fermentas, Thermo Fisher Scientific; and Oligo dT primers and QIAzol from QIAGEN Co.

### 2.2 Preparation of *Glycyrrhiza glabra* root extract

*Glycyrrhiza glabra* roots were identified by a botanist from the College of Science, Taif University, Saudi Arabia. The roots were powdered and mixed in hydro‐alcoholic solution for 24 hr at 50°C (1.0 g licorice powder extracted with 50 ml ethanol/water (30:70, v/v)). The resulting mixture was filtered using Whatman filter paper #1 and evaporated at 40°C using a rotary evaporator. The residue was stored at 4°C until use.

### 2.3 Ethics statement

The procedures and protocols used in this study were designed in accordance with the guidelines for the animal welfare and use of animals prepared by the Institutional Animal Care and Ethical Committee of Taif University, Saudi Arabia, who also gave ethical approval (# TURSP‐2020/71). All animals were treated gently to minimize suffering.

### 2.4 Animals and experimental design

28 BALB/c mice (male) were purchased from King Fahd Medical Research Center, Saudi Arabia, and were handled for a few days to help them overcome handling and treatment stress. They were split into four groups as follows: (a) control—kept at room temperature (22°C), given food and water ad libitum; (b) *G. glabra*—as above but also given *G. glabra* root extract, 200 mg/kg bw orally for 12 days; (c) gentamicin—as control, but given gentamicin, 100 mg/kg bw intraperitoneally for 12 days; and (d) gentamicin plus *G. glabra*, administered the same dosages as groups 2 and 3. Following the experimental period, the mice were anesthetized using isoflurane and then euthanized by cervical dislocation. Kidney tissue was extracted and preserved in QIAzol for RNA extraction, and 10% buffered formalin for histological and immunohistochemical analysis. Blood samples were taken and serum was extracted and stored at −20°C.

### 2.5 Biochemical assays

Blood urea nitrogen, creatinine, and urea levels in serum were measured using a colorimetric spectrophotometer, while SOD, GPX, and enzyme activity were measured following instructions.

### 2.6 Biochemical measurements of IL‐1 and IL‐6

Cytokine levels in serum were determined using sandwich ELISA kits for mouse interleukin 1 (IL‐1) beta (cat. No. E‐EL‐M0037) and IL‐6 (E‐EL‐M0044), according to the instruction manual.

### 2.7 Quantitative real‐time PCR and gene expression

Total kidney RNA was extracted and determined to be pure at 260 nm. This was used to synthesize cDNA using MyTaq Red Mix (Bioline). Gene amplification was carried out using SYBR Green Master Mix (Thermo Scientific), and the primers used are listed in Table 1. The 2^−ΔΔCt^ analysis method was used for data analysis, using the CFX96 Touch™ Real‐Time Polymerase chain reaction (PCR; Bio‐Rad Co.). Changes in gene expression and intensity were determined using Comparative Cycle Threshold (Ct) levels, normalized to β‐actin.

**TABLE 1:** Mouse Primer sequences used for quantitative real‐time polymerase chain reaction in the kidney

Gene	Accession number	Product size (bp)	Direction	Primer sequence
Nrf2	NM_010902.4	140	Sense	CGCCTGGGTTCAGTGACTCG
			Antisense	AGCACTGTGCCCTTGAGCTG
HO‐1	NM_010442.2	126	Sense	CGCCTCCAGAGTTTCCGCAT
			Antisense	GACGCTCCATCACCGGACTG
β‐actin	NM_007393.5	140	Sense	CCAGCCTTCCTTCTTGGGTA
			Antisense	CAATGCCTGGGTACATGGTG

### 2.8 Histopathology and immunohistochemistry of kidney

Kidney tissue samples were fixed, dehydrated, and set into paraffin before being sliced at 4 µm and added to slides stained with hematoxylin and eosin (H&E). The samples were examined and photographed for histopathological analysis using a Nikon Eclipse 80i microscope and a Canon SX620 H 20 megapixel digital camera (Awad et al., 2018). Immunohistochemical analysis was performed by embedding the slices in paraffin, rehydrating these, then soaking in H_2_O_2_ (2%) for 15 m, then inhibiting the peroxidase activity using PBS. A 5% bovine serum albumin was used to block nonspecific binding sites. Dilutions of 1:500 Bcl‐2‐associated X protein (Bax) Antibody (Catalog # sc‐23959), cyclooxygenase‐2 (Cox‐2) antibody (Catalog # sc‐19999), and polyclonal antibodies (Santa Cruz Biotechnology) were added to the slides and incubated at 4°C overnight. After washing the slides three times with PBS, a biotin‐conjugated secondary antibody (1:2000 dilution, cat# sc‐2040) was added. After developing the reaction with 3,3‐diaminobezidine tetrahydrochloride, the slides were counterstained using hematoxylin and the number of positively stained cells was compared to the total number of cells to determine how many of those cells were immunoreactive for Bax and Cox 2 (Nassan et al., 2018). Significance was determined using ANOVA for three different samples per group.

### 2.9 Data analysis

Seven (all) mice per group were used for data analysis. Duncan's post hoc descriptive test and one‐way ANOVA were performed using SPSS software v20.0. The data are described as means ± *SE*; statistical significance is *p* <.05.

## 3 RESULTS

### 3.1 Impact of *Glycyrrhiza glabra* root extract on quantitative renal expression of nuclear factor erythroid 2‐related factor 2 and heme oxygenase 1 genes

**FIGURE 1:**
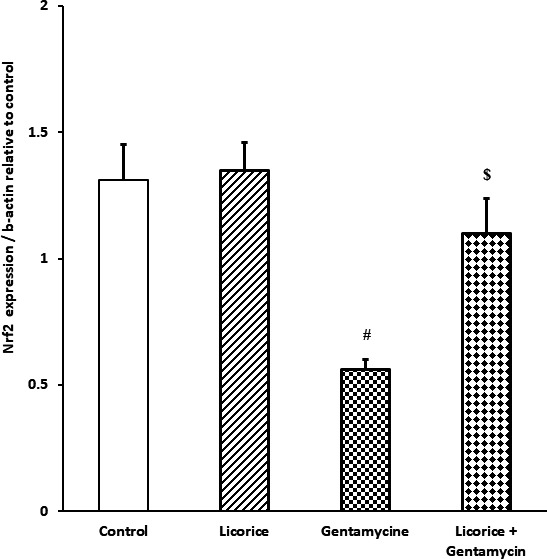
Real‐time polymerase chain reaction expression of Nrf2 in different groups relative to control. Values are means ± standard error (*SEM*) for each treatment group (*n* = 7). Values are statistically significant at ^#^ *p* <.05 versus control and *Glycyrrhiza glabra* groups, and at ^$^ *p* <.05 versus gentamicin

**FIGURE 2:**
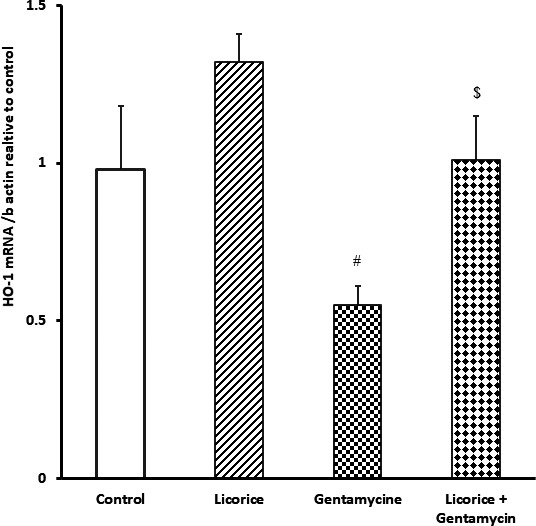
Real‐time polymerase chain reaction expression of HO‐1 in different groups relative to control. Values are means ± standard error (*SEM*) for each treatment (*n* = 7). Values are statistically significant at ^#^ *p* <.05 versus control and *Glycyrrhiza glabra* groups, And at ^$^ *p* <.05 versus gentamicin

Gentamicin‐intoxicated mice exhibited decreased expression of Nrf‐2 and heme oxygenase 1 (HO‐1) mRNA and hence increased oxidative stress in comparison with the *G. glabra*‐only and control groups. Mice that had received *G. glabra* alone showed increased nuclear factor erythroid 2‐related factor 2 (Nrf2) and HO‐1 expression, while mice that had received both gentamicin and *G. glabra* extract exhibited normal Nrf2 and HO‐1 expression (see Figures 1 and 2).

### 3.2 Serum biochemical analysis

Elevated renal biomarkers indicated kidney damage in the gentamicin‐only intoxicated group. These included serum urea (67.01 ±4.5 mg/dl, compared with 24.3 ± 1.3 control and 22.9 ± 1.4 *G. glabra*); BUN (29.4 ± 0.3 mg/dl, compared with 13.6 ± 1.9 control and 11.6 ± 0.6 *G. glabra*); and creatinine (2.1 ± 0.2 mg/dl, compared with 0.7 ± 0.02 control and 0.8 ± 0.02 *G. glabra*). These levels were all restored to normal in the group that was cotreated with *G. glabra*: serum urea returned to 31.2 ± 2.4 mg/dl, BUN to 14.9 ± 2.4 mg/dl, and creatinine to 0.9 ± 0.02 mg/dl (see Table 2).

**TABLE 2:** Effects of *Glycyrrhiza glabra* on kidney function biomarkers

	Urea (mg/dl)	BUN (mg/dl)	Creatinine (mg/dl)
Control	24.3 ± 1.3	13.6 ± 1.9	0.7 ± 0.02
*G. glabra*	22.9 ± 1.4	11.6 ± 0.6	0.8 ± 0.02
Gentamycin	67.01 ± 4.5^#^	29.4 ± 0.3^#^	2.1 ± 0.2^#^
*G. glabra* + Gentamycin	31.2 ± 2.4^$^	14.9 ± 2.4^$^	0.9 ± 0.02^$^

Note

Values are means ± standard error (*SEM*) for each treatment group (*n* = 7). Values are statistically significant at ^#^ *p* <.05 versus. control and *G. glabra* groups. ^$^ *p* <.05 versus gentamycin.

### 3.3 Effect of *Glycyrrhiza glabra* on serum antioxidant levels

The gentamicin‐only group exhibited decreased SOD and GPX, signifying oxidative stress. The *G. glabra*‐only group exhibited increased levels of GPX only, while the group cotreated with gentamycin and *G. glabra* exhibited only small changes in SOD and GPX, suggesting a restorative effect of *G. glabra* (see Table 3).

**TABLE 3:** Effects of *Glycyrrhiza glabra* extract on serum antioxidant levels

	Glutathione peroxidase (U/L)	Superoxide dismutase (U/ml)
Control	190 ± 40.5	3.3 ± 0.1
*G. glabra*	280.3 ± 35.7	3.1 ± 0.4
Gentamycin	131.2 ± 9.5^#^	2.1 ± 0.3^#^
*G. glabra* + Gentamycin	176.2.6 ± 20.9^$^	2.9 ± 0.4^$^

Note

Values are means ± standard error (*SEM*) for each treatment group (*n* = 7). Values are statistically significant at ^#^ *p* <.05 versus control and *G. glabra* groups. ^$^ *p* <.05 versus gentamycin.

### 3.4 Effect of *Glycyrrhiza glabra* on cytokine levels

Cytokine levels were elevated in the gentamicin‐only group (308.01 ± 15 pg/ml for interleukin 1 beta [IL‐1β] and 200.5 ± 16.5 pg/ml for IL‐6), compared to the control (155.5 ± 8.1 pg/ml for IL‐1β and 61 ± 6.5 pg/ml for IL‐6) and *G. glabra* groups (177.9 ± 10.5 pg/ml for IL‐1β and 81.5 ± 8.5 pg/ml for IL‐6). However, mice that had been cotreated with gentamicin and *G. glabra* exhibited normal cytokine levels (158.6 ± 26.6 pg/ml for IL‐1β and 90 ± 6.9 pg/ml for IL‐6, Table 4).

**TABLE 4:** Effects of *Glycyrrhiza glabra* extract on cytokine levels

	IL‐1β (pg/ml)	IL‐6 (pg/ml)
Control	155.5 ± 8.1	61 ± 6.5
*G. glabra*	177.9 ± 10.5	81.5 ± 8.5
Gentamycin	308.01 ± 15^#^	200.5 ± 16.5^#^
*G. glabra* + Gentamycin	158.6 ± 26.6^$^	90 ± 6.9^$^

Note

Values are means ± standard error (*SEM*) for each treatment group (*n* = 7). Values are statistically significant at ^#^ *p* <.05 versus control and *G. glabra* groups. ^$^ *p* <.05 versus gentamycin.

### 3.5 Results of histopathological examination

**FIGURE 3:**
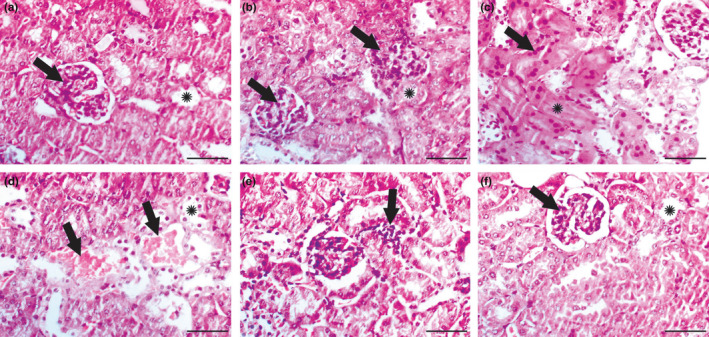
Results of histopathological examination. (a) Kidney of control group showing normal glomeruli (arrow) and tubules (*). (b) Kidney of *Glycyrrhiza glabra* group showing normal histology of both glomeruli (arrows) and tubules (*). (c–e) Kidney of gentamicin group: (c) Extensive coagulative necrosis of renal tubules was detected (*) with numerous pyknotic nuclei (arrow). (d) Severe congestion of renal blood vessels (arrows) along with hydropic degeneration of renal epithelium (*) were detected. (e) Arrow showed interstitial round cell infiltration. (f) Gentamicin + *Glycyrrhiza glabra* group showed restoration of normal glomerular (arrow) and tubular (*) architecture. Scale bar = 50 μm

Mice in the control group exhibited normal cortex and medulla, and normal tubular and glomerular architecture (Figure 3a). This histological profile was the same for the *G. glabra*‐only group (Figure 3b). The kidney of gentamicin group mice showed severe damage, including coagulative necrotic changes; while the tissue architecture remained intact, cellular details were lost (Figure 3c). Further, the gentamicin group exhibited severe renal blood vessel congestion, as well as hydropic degeneration of renal tubular epithelium (Figure 3d) and focal interstitial round cell aggregation (Figure 3e). A normal histological profile was observed in the cotreated gentamicin and *G. glabra* group (Figure 3f).

### 3.6 Results of immunohistochemical examination of Cox‐2 and Bax expression

**FIGURE 4:**
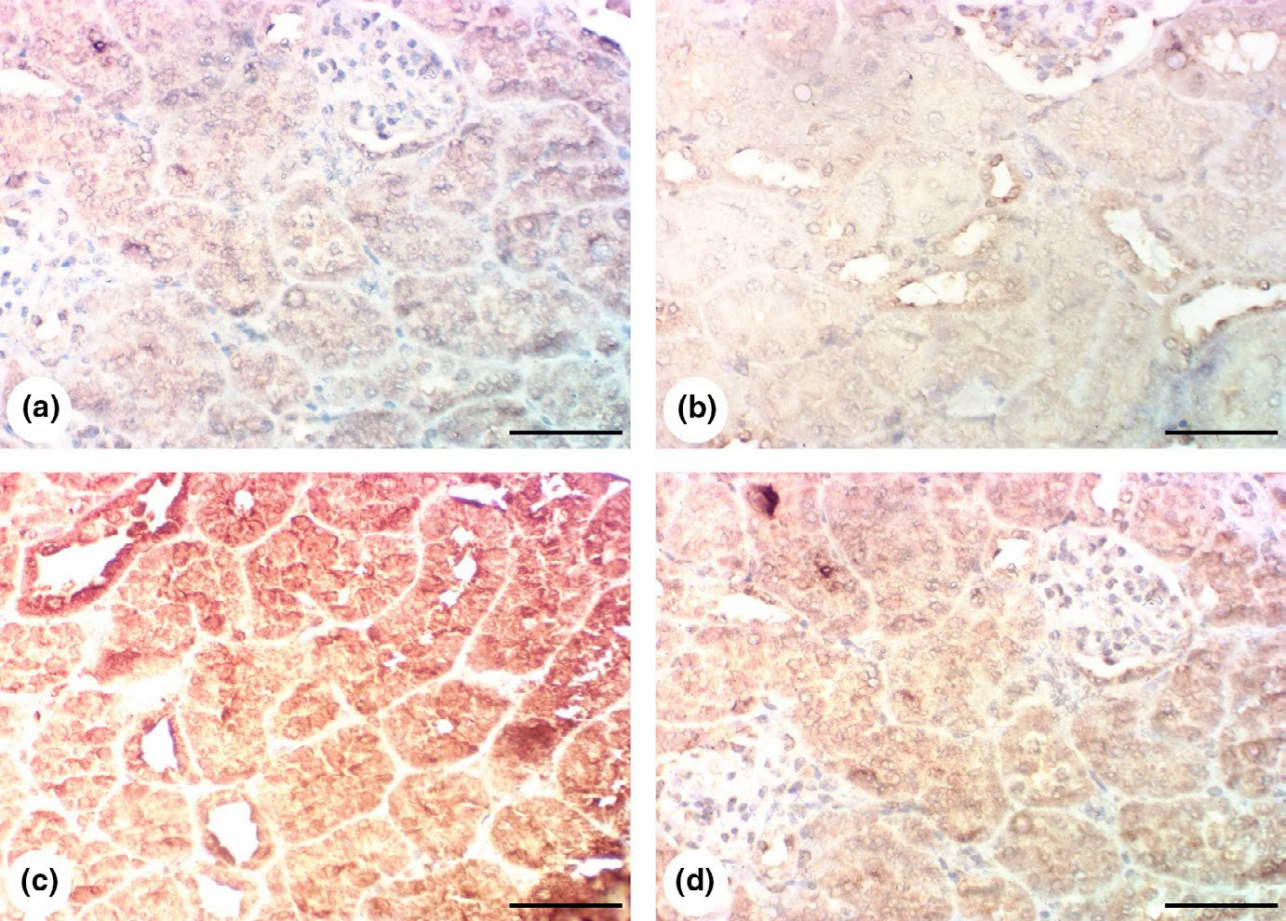
Results of immunohistochemical examination of Cox‐2: (a, b) Kidneys of control and *Glycyrrhiza glabra* groups showing poor expression of Cox‐2 in renal tissue. (c) Kidney of gentamicin group showing overexpression of Cox‐2 in renal tissue. (d) Gentamicin + *G. glabra* group showing reduction of Cox‐2 expression. Scale bar = 50 μm

Kidney tissue samples from both the control and *G. glabra*‐only groups exhibited low Cox‐2 expression (Figure 4a,b). However, Cox‐2 expression was elevated in the renal tubules in the gentamicin‐only group (Figure 4c), while the *G. glabra* cotreated group exhibited a reduction in cox‐2 expression (Figure 4d).

**FIGURE 5:**
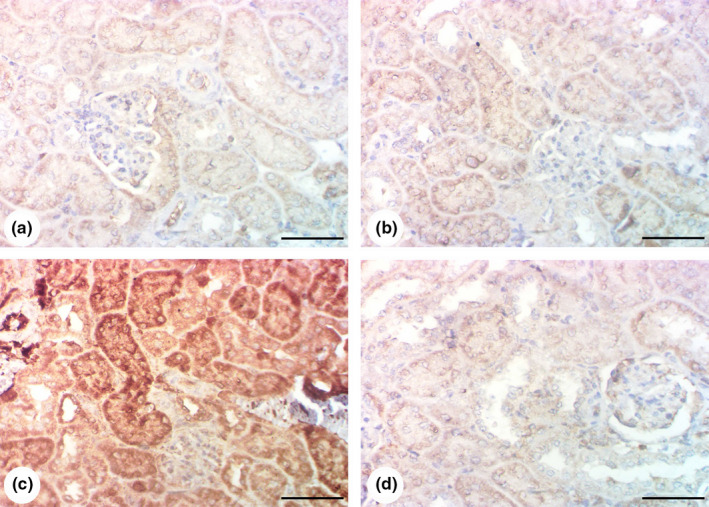
Results of immunohistochemical examination of Bax: (a, b) Kidney of control and *Glycyrrhiza glabra* group showing poor expression of Bax in renal tissue. (c) Kidney of gentamicin group showing increased Bax expression in renal tissue. (d) Gentamicin + *G. glabra* group showing a decline of Bax expression. Scale bar = 50 μm

Similarly, the control and *G. glabra*‐only samples showed low Bax expression (Figure 5a,b). Bax expression was raised in the gentamicin‐only group (Figure 5c) and significantly decreased in the cotreated *G. glabra* group (Figure 5d).

## 4 DISCUSSION

Aminoglycoside antibiotics are highly effective in the treatment of gram‐negative bacterial infections, and gentamicin is the most commonly used of this type of antibiotic (Lopez‐Novoa et al., 2011). Unfortunately, gentamicin also has serious side effects, with nephrotoxicity reaching up to 50% over a 14‐day course of therapy (Leehey et al., 1993), meaning that its use must be carefully controlled. The damage caused by gentamicin increases morbidity and, more specifically, hydropic degeneration and congestion of renal blood vessels, and coagulative necrosis of tubular epithelium, as observed in the present study. Functional and necrotic changes of cellular components leads to tubular damage (Randjelovic et al., 2017), as well as disruption to urinary concentration and ultimately, acute renal failure. As a result—and as observed in this study, serum levels of urea, BUN, and creatinine increase (Hajihashemi et al., 2020). Gentamicin is also linked with tubular epithelial cell necrosis (Edwards et al., 2007) and apoptosis (El Mouedden et al., 2000; Li et al., 2009). Nephrotoxicity is evident in the epithelial cells, through the endocytic pathway, and also in renal proximal tubules, causing tubular injury, leading to epithelial apoptosis and necrosis (Martínez‐Salgado et al., 2007; Nagai & Takano, 2014; Randjelovic et al., 2017). The multi‐ligand binding receptor, megalin, which can be found in the apical brush border of the renal proximal tubular epithelial cells is involved in gentamicin uptake and renal accumulation (Nagai et al., 2001). Once bound and internalized through endocytosis, gentamicin travels to the endoplasmic reticulum, Golgi apparatus, and lysosomes, causing a build‐up of phospholipids, impacting on lipid metabolism, ultimately leading to apoptosis (Martínez‐Salgado et al., 2007).

The gentamicin‐only group exhibited elevated Bax expression in kidney samples. It is speculated that this was caused by membrane rupture, releasing gentamicin into the cytoplasm. In turn, this triggers pro‐apoptotic proteins, which damage mitochondria, leading to the release of reactive oxygen species (ROS) and consequent inflammation (Jado et al., 2020).

Acute kidney damage is often treated using *G. glabra*, which stimulates increased aldosterone and cortisone production from the adrenal cortex—hormones, which alleviate stress and exhaustion (Aksoy et al., 2012). *Glycyrrhiza glabra* has long been used in traditional medical contexts, especially Chinese medicine, in the treatment of kidney‐related disorders (Aksoy et al., 2012), and its nephroprotective effects are well‐established. Studies have identified *G. glabra*'s protective effect to be related to its antioxidant properties (Ju et al., 2017), which is in line with this study's finding of GPX and SOD restoration following *G. glabra* administration. Similarly, the gentamicin‐intoxicated group exhibited a decline in HO‐1 and Nrf2, which was restored in the *G. glabra* group. As an endogenous antioxidant, HO‐1 plays a key role in the defense against oxidation. Nrf2 is also critical in muting the oxidative stress response, through regulating antioxidant protein expression (Ding et al., 2020). Nrf2 targets the HO‐1 gene, which is responsible for eliminating damaged mitochondria through autophagy and thereby inhibiting one of the major causes of oxidative stress (Unuma et al., 2013). The present study indicated that *G. glabra* promotes HO‐1 and Nrf2 expression, thereby increasing antioxidant responses.

The dosage and treatment length for the intoxicated mice were based on previous studies that had found nephrotoxicity to be successfully induced by100 mg/kg bw gentamicin for 8 days (Hussain et al., 2012; Saleem et al., 2019; Sun et al., 2018; Whiting et al., 1981). Inflammation in the kidney reduces clearance rates and in particular makes urinary clearance less efficient, causing toxic molecules and drugs to accumulate. Inflammation is regulated by Cox‐2 (Minghetti, 2004) which, stimulated by pro‐inflammatory cytokines, spreads around the kidney during inflammatory responses, ultimately causing renal damage (Deng et al., 2020). Mice in the gentamicin groups exhibited increased Cox‐2 levels, indicating a dysregulated inflammation response, likely to result in kidney damage. The group that was co‐administered with *G. glabra* showed a reduction in Cox‐2. A primary function of Cox‐2 is to promote PGE_2_ generation, which exacerbates inflammatory responses under certain stimuli (Smith, 1989).

The findings in this study indicate *G. glabra*'s protective effect against inflammatory damage to the renal tubular cells. *Glycyrrhiza glabra*'s anti‐inflammatory effect was shown through Cox‐2, IL‐1β, and IL‐6 measurements, which had an anti‐apoptotic effect under intoxication intoxication. Cox‐2 activity was inhibited by *G. glabra*. In turn, arachidonic acid and prostaglandin, which normally lead to platelet aggregation, increased temperature, and inflammation (Vitale et al., 2016), were reduced. A number of other studies have found that Cox‐2 expression can be inhibited by naturally occurring herbs (Liang et al., 2001), which regulate nitric oxide production. When superoxide anions are present, these react with nitric oxide, producing peroxynitrite, a powerful oxidizing agent (Beckman & Koppenol, 1996). Studies elsewhere have already identified that gentamicin‐induced renal intoxication is associated with elevated nitric oxide levels (Rivas‐Cabañero et al., 1997).

From this, we infer that the most likely mechanism by which *G. glabra* exerts a renal‐protective effect is through increasing SOD and Gpx serum levels; promoting the expression of Nrf2 and Cox‐2, resulting in anti‐inflammatory responses; and inhibiting IL‐1β and IL‐6 production. The anti‐apoptotic response can be linked to the phenolic and flavonoid compounds present in the herb.

## 5 CONCLUSIONS

*Glycyrrhiza glabra* is recommended as a complimentary renoprotective treatment, especially with nephrotoxic drugs such as gentamicin, due to its anti‐inflammatory, antioxidant, cytoprotective, and anti‐apoptotic properties.

## CONFLICT OF INTEREST

All authors declare that they do not have any conflict of interest that could inappropriately influence this manuscript.

## ETHICS APPROVAL

The ethical committee at Turabah University College approved all experimental procedures in accordance with the institutional guidelines for the Care and Use of Laboratory Animals (Project number TURSP‐2020/71).

## Data Availability

The materials and data included within the study are available from the corresponding author upon reasonable request.
